# Appraisal of Cinnamaldehyde Analogs as Dual-Acting Antibiofilm and Anthelmintic Agents

**DOI:** 10.3389/fmicb.2022.818165

**Published:** 2022-03-16

**Authors:** Sagar Kiran Khadke, Jin-Hyung Lee, Yong-Guy Kim, Vinit Raj, Jintae Lee

**Affiliations:** School of Chemical Engineering, Yeungnam University, Gyeongsan, South Korea

**Keywords:** antibiofilm, anthelmintic, *Candida albicans*, α-methyl cinnamaldehyde, *trans*-4-methyl cinnamaldehyde, protein interaction

## Abstract

Cinnamaldehyde has a broad range of biological activities, which include antibiofilm and anthelmintic activities. The ever-growing problem of drug resistance and limited treatment options have created an urgent demand for natural molecules with antibiofilm and anthelmintic properties. Hence, we hypothesized that molecules with a scaffold structurally similar to that of cinnamaldehyde might act as dual inhibitors against fungal biofilms and helminths. In this regard, eleven cinnamaldehyde analogs were tested to determine their effects on fungal *Candida albicans* biofilm and nematode *Caenorhabditis elegans*. α-Methyl and *trans*-4-methyl cinnamaldehydes efficiently inhibited *C. albicans* biofilm formation (>90% inhibition at 50 μg/mL) with minimum inhibitory concentrations (MICs) of ≥ 200 μg/mL and 4-bromo and 4-chloro cinnamaldehydes exhibited anthelmintic property at 20 μg/mL against *C. elegans*. α-Methyl and *trans*-4-methyl cinnamaldehydes inhibited hyphal growth and cell aggregation. Scanning electron microscopy was employed to determine the surface architecture of *C. albicans* biofilm and cuticle of *C. elegans*, and confocal laser scanning microscopy was used to determine biofilm characteristics. The perturbation in gene expression of *C. albicans* was investigated using qRT-PCR analysis and α-methyl and *trans*-4-methyl cinnamaldehydes exhibited down-regulation of *ECE1*, *IFD6*, *RBT5*, *UCF1*, and *UME6* and up-regulation of *CHT4* and *YWP1*. Additionally, molecular interaction of these two molecules with UCF1 and YWP1 were revealed by molecular docking simulation. Our observations collectively suggest α-methyl and *trans*-4-methyl cinnamaldehydes are potent biofilm inhibitors and that 4-bromo and 4-chloro cinnamaldehydes are anthelmintic agents. Efforts are required to determine the range of potential therapeutic applications of cinnamaldehyde analogs.

## Introduction

Plants are one of the prime sources of bioactive molecules. Cinnamaldehydes are present in the bark of trees of the genus *Cinnamomum*, which contains around 250 plant species (Shreaz et al., 2016). Highest percentages of cinnamaldehyde are found in two common species, namely, *Cinnamomum cassia* and *Cinnamomum verum* (also called *C. zeylanicum*) (Chen et al., 2017; Doyle and Stephens, 2019). *Trans*-cinnamaldehyde the predominant form in cinnamon is a phenylpropanoid and is generally recognized as safe by United States Food and Drug Administration (USFDA), and the Flavor and Extract Manufacturer’s Association (FEMA), and the Council of Europe has given it an A status for use in foodstuffs (Friedman, 2017). *Trans*-cinnamaldehyde is a yellow oil with a sweet taste and the odor of cinnamon and is primarily used as a flavoring agent as well as used in medical products, cosmetics, and perfumes (Brackman et al., 2008; Doyle and Stephens, 2019). Furthermore, *trans*-cinnamaldehyde has also been documented to have antibiofilm and anti-quorum sensing activity against *Vibrio harveyi* (Niu et al., 2006), enterohemorrhagic and uropathogenic *Escherichia coli* strains (Kim et al., 2015; Kot et al., 2015), methicillin-resistant *Staphylococcus aureus* strains (Kavanaugh and Ribbeck, 2012; Kot et al., 2018), *Pseudomonas aeruginosa* (Kavanaugh and Ribbeck, 2012), *Pseudomonas fluorescens* (Li et al., 2018), *Cronobacter sakazakii* (Amalaradjou and Venkitanarayanan, 2011), *Streptococcus pyogenes* (Beema Shafreen et al., 2014), *Salmonella typhimurium* (Silva et al., 2018), and against the pathogenic fungus *C. albicans* (Ying et al., 2019; Miranda-Cadena et al., 2021). In addition, cinnamaldehyde analogs are known to have an array of bioactivities, which include antibacterial (Firmino et al., 2018), antifungal (Da Nobrega Alves et al., 2020), antiviral (Hayashi et al., 2007), antiulcer (Tabak et al., 1999), antioxidant (Mathew and Abraham, 2006), antidiabetic (Im et al., 2014), anti-inflammatory (Srisook et al., 2019), anticancer (Fang et al., 2004) activities and insecticidal (Cheng et al., 2009; Lu et al., 2020), larvicidal (Cheng et al., 2004), nematicidal (Ferreira Barros et al., 2021), and anthelmintic (Williams et al., 2015) effects.

The failure of current antifungal treatments caused by their overuse and the consequent emergence of multidrug-resistant variants of microorganisms presents a challengeable problem (Cegelski et al., 2008; Liu et al., 2020; Lin et al., 2021). Bacteria and fungi protect themselves from antimicrobial agents, host defense systems, and nutrient limitations by forming self-organized and three-dimensional communities (biofilms) on various biotic or abiotic surfaces (Costerton et al., 1999; Tan et al., 2019), and by so doing contribute to the persistence of infections. Conventional antifungal agents inhibit planktonic fungal growth, which often results in drug resistance (Hentzer et al., 2002; Ma et al., 2020). A report published in 2019 by the Centers for Disease Control and Prevention (CDC, 2019) stated that *Candida albicans* presents a serious threat *via* the spread of life-threatening candidiasis (Ostrosky-Zeichner et al., 2010; CDC, 2019). *C. albicans* and its biofilms are found on mucosal surfaces and in the gastrointestinal and genitourinary tracts, and *C. albicans* readily colonizes host tissues and indwelling medical devices such as urinary catheters, dental implants, artificial heart valves, joint prosthetics, penile implants, and intrauterine devices (Ramage et al., 2005; Sardi et al., 2013; De Oliveira et al., 2019; Handorf et al., 2019). Highly structured *C. albicans* biofilms form on implant surfaces and subsequently the pathogen disseminate into blood to cause invasive candidiasis, which is responsible for an estimated 100,000 deaths per annum in the United States and for the replacement of over five million central nervous catheters (Sardi et al., 2013). Highly resistant fungal biofilm infections are treated using high antifungal doses and the removal of colonized medical devices, which pose risks of kidney and liver damage and substantially increase medical costs (Ramage et al., 2005; De Oliveira et al., 2019).

On the other hand, parasitic nematodes have serious impacts on global health and socio-economic development, for example, several soil-transmitted nematodes directly affect human populations, pose major threats to livestock and plants, and are responsible for huge economic losses (Hotez et al., 2014). Unfortunately, the originally limited armamentarium of anthelmintic drugs and their extensive usage have directed to drug resistance, and thus, there is an urgent need to discover new drug candidates (Diawara et al., 2013; Kotze et al., 2014; Krücken et al., 2017). The evaluation of the anthelmintic efficacies of candidate compounds against parasitic nematodes poses several challenges, which include limited access to lifecycle stages, cost-intensive laboratory studies on life cycles and host dependency, and fragile and complex *in vitro* culture (Burns et al., 2015; Hahnel et al., 2020). Free-living, transparent *Caenorhabditis elegans* has been well used as a model system for evaluation purposes. This nematode has a simple, rapid life cycle and well-annotated genes, and an extensive number of molecular tools (Brenner, 1974). *C. elegans* shares the typical anatomical characteristics of most nematode species as regards its body plan, cuticle, and nervous system organization (Harder, 2016).

**SCHEME 1:**
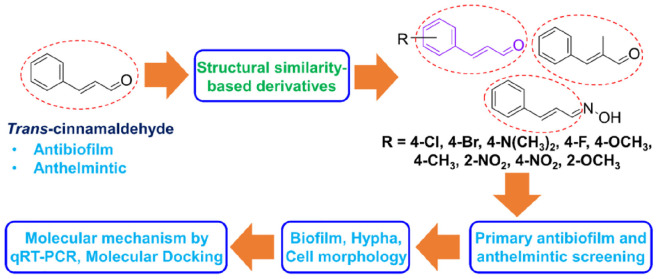
Schematic representation of the strategies used in the present study.

Inspired by the broad-ranging biological activities of the cinnamaldehyde scaffold including antibiofilm and anthelmintic potencies as well as safety profile of naturally isolated cinnamaldehyde and the current challenges posed by drug-resistant fungi and parasites, hence it can be hypothesized that cinnamaldehyde analogs may be good alternative against *C. albicans* biofilm and nematode *C. elegans*. To check this hypothesis, we selected cost-effective eleven cinnamaldehyde analogs for the screening *in vitro* antibiofilm and *in vivo* anthelmintic activities. In order to explore surface morphological effect of two active cinnamaldehyde analogs on *C. albicans* biofilm and *C. elegans*, scanning electron microscopy (SEM) was carried out. Also, confocal laser scanning microscopy (CLSM) was utilized for biofilms of *C. albicans*. Further, the antibiofilm effects of the two most active cinnamaldehyde analogs, α-methyl cinnamaldehyde and *trans*-4-methyl cinnamaldehyde, were investigated using hyphae formation and cell aggregation assays. The molecular mechanism of α-methyl cinnamaldehyde and *trans*-4-methyl cinnamaldehyde was estimated using quantitative real-time PCR (qRT-PCR) analysis and molecular interaction of α-methyl cinnamaldehyde and *trans*-4-methyl cinnamaldehyde with proteins UCF1 (filament specific regulator) and YWP1 (yeast form wall protein 1) was predicted by molecular simulation (Scheme 1). To the best of our knowledge, this is the first report on the antibiofilm and anthelmintic activities of cinnamaldehyde analogs against *C. albicans* and *C. elegans*, respectively.

## Materials and Methods

### Reagents and Culture Strains

**FIGURE 1:**
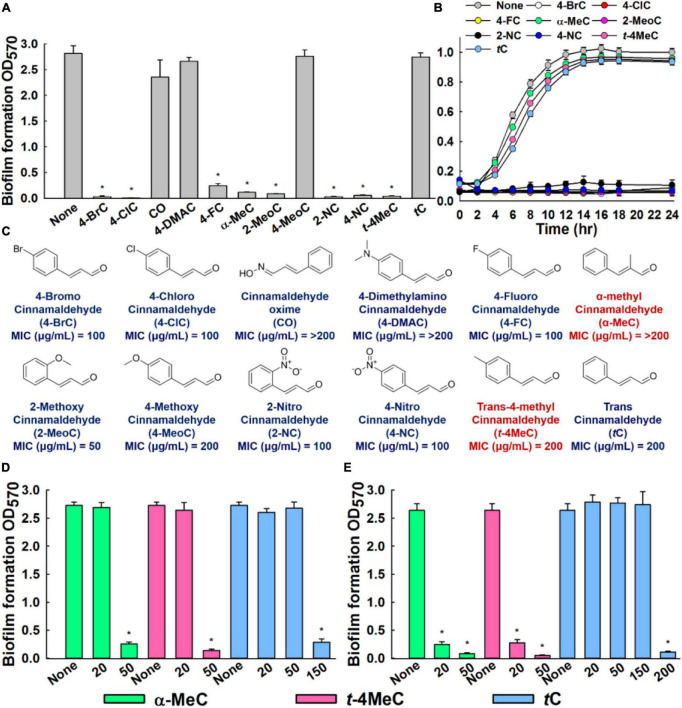
*In vitro* antibiofilm activities of cinnamaldehyde analogs against *C. albicans*. Influences of eleven cinnamaldehyde analogs and *trans*-cinnamaldehyde (*t*C) on biofilm formation by the *C. albicans* DAY185 at 100 μg/mL **(A)**, *C. albicans* DAY185 cell growth was investigated in the presence of the eight antibiofilm cinnamaldehyde analogs and the *trans*-cinnamaldehyde control at 100 μg/mL **(B)**, Chemical structures and MIC **(C)**. The antibiofilm activities of α-methyl cinnamaldehyde (α-MeC) and *trans*-4-methyl cinnamaldehyde (*t*-4MeC) at 20 and 50 μg/mL, and *trans*-cinnamaldehyde (*t*C) at 20–200 μg/mL against *C. albicans* DAY185 **(D)** and *C. albicans* ATCC 10231 **(E)**. Error bars indicate standard deviations. *P < 0.05 vs. non-treated controls (None).

Chemicals including eleven cinnamaldehyde analogs viz. 4-bromo cinnamaldehyde (95%), 4-chloro cinnamaldehyde (95%), cinnamaldehyde oxime (95%), 4-dimethylamino cinnamaldehyde (98%), 4-fluoro cinnamaldehyde (97%), α-methyl cinnamaldehyde (95%), 2-methoxy cinnamaldehyde (95%), 4-methoxy cinnamaldehyde (95%), 2-nitro cinnamaldehyde (98%), 4-nitro cinnamaldehyde (95%), *trans*-4-methyl cinnamaldehyde (95%), and one positive control; *trans*-cinnamaldehyde (99%) (Figure 1C), dimethyl sulfoxide (DMSO) (99%) and crystal violet (90%) were purchased from either Sigma-Aldrich (St. Louis, MO, United States), Combi Blocks, Inc., (San Diego, CA, United States) or TCI Co., (Tokyo, Japan). The fluconazole-resistant *C. albicans* strains DAY185 and ATCC 10231 used were obtained from the Korean Culture Center for Microorganisms (Seoul, South Korea) (Lee et al., 2018). For the experiments, *C. albicans* strains DAY185 and ATCC 10231 were cultured under aerobic conditions at 37°C in potato dextrose agar (PDA; Becton Dickinson, Sparks, MD, United States) and potato dextrose broth (PDB; Becton Dickinson, Sparks, MD, United States). Initially, fungal strains were taken from −80°C glycerol stock and streaked onto potato dextrose agar plates. Single fresh colonies were inoculated into PDB (2 mL) in 14 mL round-bottom tubes and incubated at 37°C at 250 rpm (Lee et al., 2018). Cinnamaldehyde analogs were dissolved in required quantities of DMSO. DMSO [0.1% (v/v)] was used as the negative control and this concentration did not inhibit fungal growth or biofilm formation.

### Biofilm Formation, Minimum Inhibitory Concentrations, and Growth Rate Measurements for the Screening of Cinnamaldehyde Analogs Against *C. albicans*

Biofilm formation assays were performed in 96-well microtiter plates using the crystal violet staining method, as previously described (Lee et al., 2021). Briefly, a 2-day-old single colony of *C. albicans* was inoculated into PDB and incubated overnight at 37°C with shaking. Overnight cultures of initial turbidity 0.1 at OD_600_ nm (∼10^5^ CFU/mL) were re-inoculated into fresh PDB (final volume 300 μL) and concurrently treated individually with or without the presence of eleven cinnamaldehyde analogs at 100 μg/mL and *trans*-cinnamaldehyde. Microtiter plates were incubated at 37°C without shaking for 24 h, and biofilms that adhered to plate bottoms were stained with 0.1% crystal violet for 20 min, repeatedly washed with sterile distilled water, and resuspended in 95% ethanol. Plates were read at OD_570_ nm to assess biofilm formation using a Multiskan EX microplate reader (Thermo Fisher Scientific, Waltham, MA, United States). Besides, the minimum inhibitory concentrations (MICs) of cinnamaldehyde analogs were determined as per Clinical and Laboratory Standards Institute (CLSI) guidelines (CLSI, 2017). Overnight cultures of *C. albicans* were treated with each cinnamaldehyde analog at various concentrations (0–200 μg/mL) and incubated at 37°C for 24 h. MIC was defined as the lowest concentration that inhibited yeast growth by 80%, as assessed by spectrophotometry (620 nm). The results quoted are the averages of at least two independent cultures.

Based on outcomes of biofilm experiment, cell growth analysis was carried out as follows. *C. albicans* DAY185 was re-inoculated into 96-well plates containing PDB medium (1:50 dilution) and treated with or without the eight most potent cinnamaldehyde analogs, as determined by biofilm formation assay, that is, 4-bromo, 4-chloro, 4-fluoro, α-methyl, 2-methoxy, 2-nitro, 4-nitro, *trans*-4-methyl, or *trans*-cinnamaldehydes at 50–100 μg/mL for 24 h at 37°C. Afterward, *C. albicans* ATCC 10231 was inoculated into 96-well plates containing PDB medium (1:50 dilution) and treated with or without two most potent cinnamaldehyde analogs at 50–100 μg/mL for 24 h at 37°C. Growths were assessed by spectrophotometry at OD_620_. Consequently, two cinnamaldehyde analogs were selected based on their antibiofilm potency. Later, doses dependent antibiofilm effects of highly potent cinnamaldehyde analogs were revealed at 0–50 μg/mL. Results are the averages of measurements taken from at least six replicate wells.

### Colony Morphology Assay on Solid Media

Potato dextrose agar plates containing or not α-methyl, *trans*-4-methyl, or *trans*-cinnamaldehydes (0–50 μg/mL) were streaked with *C. albicans* DAY185 or ATCC 10231 and incubated at 37°C for 6 days. Plates were periodically monitored for colony formation and morphology, and phenotypic changes were observed using the iRiS™ Digital Cell Imaging System (Logos BioSystems, South Korea) at 10x (Lee et al., 2021). At least, three independent experiments were conducted.

### Yeast-Hyphae-Transition Assay

Assays were conducted in liquid media, as previously described (Lee et al., 2021). *C. albicans* DAY185 or ATCC 10231 cells at a density of 10^5^ CFU/mL were inoculated in 2 mL of PDB medium and treated with α-methyl, *trans*-4-methyl, or *trans*-cinnamaldehydes at 0–50 μg/mL. Disposable, sterile polypropylene tubes (14 mL) with polyethylene caps were used to conduct hyphal assays in PDB. Tubes were demonstrated by the manufacturer to create aerobic (open-cap) conditions. Cultures containing cinnamaldehyde analogs or not were incubated at 37°C without shaking for 24 h, aliquoted, and imaged in bright field using the iRiS™ Digital Cell Imaging System at 20x. At least, four independent experiments were conducted.

### Biofilm Annotations by Confocal Laser Scanning Microscopy

*Candida albicans* biofilms were produced on 96-well polystyrene plates in the presence or absence of α-methyl, *trans*-4-methyl, or *trans*-cinnamaldehydes at 50 μg/mL without shaking for 24 h at 37°C. After incubation, planktonic cells were removed by washing (three times) with distilled water, and biofilms were stained with carboxyfluorescein diacetate succinimidyl ester (Invitrogen, Eugene, OR, United States) (Lee et al., 2019a). Plate bottoms were then visualized using a 488 nm Ar laser (emission 500–550 nm) beneath a confocal laser microscope (Nikon Eclipse Ti, Tokyo, Japan). To quantify biofilm structures, COMSTAT software (Heydorn et al., 2000) was used to determine biovolumes (μm^3^ μm^–2^), mean biofilm thicknesses (μm), and percentage substratum coverages (%). Two autonomous cultures were performed for each experimental condition and at least 10 random positions were screened.

### Microscopic Architecture of *C. albicans* Biofilms and *C. elegans* Cuticles

Scanning electron microscopy was used to observe biofilms, as previously described (Kim et al., 2020). Briefly, precut pieces of a nylon membrane 0.5 × 0.5 cm were placed in 96-well plates containing *C. albicans* grown in PDB medium with or without α-methyl, *trans*-4-methyl, or *trans*-cinnamaldehydes (50 μg/mL) and incubated for 24 h at 37°C. Cells that adhered to nylon membranes for 24 h were fixed with a glutaraldehyde (2.5%) and formaldehyde (2%) solution, postfixed using osmium tetroxide, and dehydrated using an ethanol series (50, 70, 80, 90, 95, and 100%) and isoamyl acetate. After critical-point drying, cells were sputter-coated with palladium/gold and imaged using an S-4200 scanning electron microscope (Hitachi, Tokyo, Japan) at 15 kV.

To examine *C. elegans* cuticles, scanning electron microscopy was performed using an S-4800 instrument (Hitachi, Tokyo, Japan), as described previously (Ropiak et al., 2016). To investigate the effects of the highly potent anthelmintic agents, nematodes were treated with 4-bromo or 4-chloro cinnamaldehydes at 20 μg/mL for 48 h, and then 10 nematodes per treatment were processed for SEM imaging, as previously described (Ropiak et al., 2016). *trans*-cinnamaldehyde was used as the control.

### RNA Isolation and qRT-PCR for Transcriptomic Profile of *C. albicans*

Transcript expression analysis was conducted using concentrate of 25 mL cultures of *C. albicans* at an initial turbidity of 0.1 at OD_600_ (∼10^5^CFU/mL). These were incubated for 6 h at 37°C with agitation (250 rpm) in the presence or absence of α-methyl or *trans*-4-methyl cinnamaldehydes (50 μg/mL). RNA degradation was prevented by adding RNase inhibitor (RNAlater, Ambion, TX, United States) to cells immediately after incubation. Total RNA was isolated using a hot acidic phenol method (Amin-Ul Mannan et al., 2009) and RNA was purified using the Qiagen RNeasy mini Kit (Valencia, CA, United States).

To determine the expressions of hyphal and biofilm-related genes (*ALS3*, *CHT4*, *ECE1*, *HWP1*, *IFD6*, *RAS1*, *RBT5*, *UCF1*, *UME6*, and *YWP1*), qRT-PCR was performed as described (Kim et al., 2016) using SYBR Green master mix (Applied Biosystems, Foster City, CA, United States) and an ABI StepOne Real-Time PCR System (Applied Biosystems). The housekeeping gene (*RDN18*) and the primers used for qRT-PCR are listed in Supplementary Table S1. At least two independent cultures were used.

### Preparation of Ligands and Computational Screening

Ligand structures were prepared using LigPrep tools in the Schrodinger suite and optimized for minimum energy using the density functional theory (DFT) approach, as described previously (Khadke et al., 2021; Raj et al., 2021). The main reason of molecular docking was to confirm and reveal the molecular interaction between potent cinnamaldehyde analogs and highly significant genes of interest. Based on the gene expression perturbation, we have chosen *UCF1* and *YWP1* owing to their prominent downregulation (12- and 54-fold change) and upregulation (26- and 17-fold change), respectively. Hence, UCF1 and YWP1 proteins were selected for docking study to confirm that whether α-methyl and *trans*-4-methyl cinnamaldehyde can interact with these proteins. Conformations and bond orders were minimized and refined using the OPLS 2005 force field. Prepared ligands were subjected to analysis for computational screening with the active binding pocket of UCF1 and YWP1. Initially, the binding active pockets of UCF1 and YWP1 were predicted by the CASTp server^1^ (Tian et al., 2018). These predicted active sites were assigned for a final grid by molecular screening by treating drug molecules as rigid entities and receptors as flexible entities. To ensure the reliability, validity, and reproducibility of docking results, molecular docking was performed using AUTODOCK (Seeliger and De Groot, 2010). Additionally, cluster analysis of these targeted molecules was carried out with UCF1 and YWP1. Further, binding energies and interactions between α-methyl, *trans*-4-methyl, or *trans*-cinnamaldehydes and UCF1 or YWP1 were determined using a computational approach, as previously described (Raj et al., 2021). BIOVIA Discovery Studio Visualizer was used to capture interactions between the cinnamaldehyde analogs and UCF1 or YWP1.

### Assessment of the *in vivo* Anthelmintic Activities of Cinnamaldehyde Analogs

*Caenorhabditis elegans fer-15(b26)*; *fem-1(hc17)* (Garigan et al., 2002) strain was obtained from Prof. Eleftherios Mylonakis (Brown University). The strain was maintained on a nematode growth medium (NGM) with *E. coli* OP50 as feed, and synchronized as previously described protocol (Lee et al., 2017). Briefly, *C. elegans* worms, eggs were collected in worm-lysis solution (2% sodium hypochlorite and 0.5 N sodium hydroxide) from adults, washed and allowed to hatch to the L1 stage in M9 buffer for 24 h at 25°C under 6 rpm rotation (Lee et al., 2017). Later, worms were transferred to fresh NGM plates containing *E. coli* OP50 lawns to obtain synchronized L4 stage worms and collected in M9 buffer, washed, and transferred to 96-well plate.

*In vivo* anthelmintic activities were investigated to confirm the anthelmintic effects of the eleven cinnamaldehyde analogs and *trans*-cinnamaldehyde using a previously described *C. elegans* model (Ropiak et al., 2016; Lee et al., 2019b). In brief, synchronized *C. elegans fer-15(b26)*; *fem-1(hc17)* worms (*n* = ∼20–30) were pipetted into the wells of a 96-well plate in M9 buffer. Cinnamaldehyde analogs (5–100 μg/mL) were then added to a final volume of 300 μL. Nematodes were incubated for 4 days at 25°C, and viabilities were determined using an iRiS™ Digital Cell Imaging System (Logos BioSystems, South Korea) by exposing worms to LED or UV LED light for 10–30 s (Rajasekharan et al., 2018). Three independent experiments were performed in triplicate.

### Estimation of Absorption, Distribution, Metabolism, and Excretion Properties of Cinnamaldehyde Analogs

The drug-likeness parameters of two most potent antibiofilm and anthelmintic cinnamaldehyde analogs and *trans*-cinnamaldehyde were evaluated using Swiss Absorption, Distribution, Metabolism, and Excretion (ADME) (Daina et al., 2017). According to the Lipinski rule, an orally active pharmaceutical agent should have a molecular weight of ≤ 500 g/moL, a Log *P* of ≤ 5, ≤ 5 hydrogen bond-donating atoms, ≤ 10 hydrogen-bond accepting atoms, and a topological polar surface of ≤ 140Å^2^ (Benet et al., 2016).

### Statistical Analysis

Replication numbers for assays are provided above, and results are presented as means ± standard deviations. The statistical analysis was performed using one-way ANOVA followed by Dunnett’s test in SPSS version 23 (SPSS Inc., Chicago, IL, United States). *P* values of < 0.05 were considered significant. Asterisks are used to denote significant differences between treated and untreated samples.

## Results

### *In vitro* Assessments of the Antibiofilm Activities of Cinnamaldehyde Analogs Against *C. albicans*

The antibiofilm potencies of the eleven cinnamaldehyde analogs at 100 μg/mL were initially investigated using *C. albicans* DAY185 and their potencies were compared with *trans*-cinnamaldehyde (Figure 1A). At initial screening, eight analogs, that is, 4-bromo, 4-chloro, 4-fluoro, α-methyl, 2-methoxy, 2-nitro, 4-nitro, and *trans*-4-methyl cinnamaldehydes displayed strong antibiofilm activities (98, 99, 91, 95, 96, 98, 97, and 98%, respectively) against *C. albicans* DAY185. In contrast, cinnamaldehyde oxime, 4-dimethylamino cinnamaldehyde, and 4-methoxy cinnamaldehyde showed no or little biofilm inhibitory activity. Among the initially screened eight antibiofilm inhibitors, six had MIC in the range of 50–100 μg/mL, while two remained cinnamaldehyde analogs showed MIC of ≥ 200 μg/mL (Figure 1C). Usually, antifungal agents inhibit microorganism planktonic growth, and this inhibition can lead to drug resistance, thus based on MIC’s and our focus relying on finding lead antibiofilm agents, we selected α-methyl and *trans*-4-methyl cinnamaldehydes for further investigations (Figures 1A,B).

In more detail, α-methyl and *trans*-4-methyl cinnamaldehydes dose-dependently inhibited biofilm formation by both *C. albicans* strains. For example, α-methyl and *trans*-4-methyl cinnamaldehydes at 50 μg/mL inhibited *C. albicans* DAY185 biofilm formations by > 90% (Figure 1D). Also, both the analogs inhibited *C. albicans* ATCC 10231 biofilm formations by > 88 and > 95%, at 20 and 50 μg/mL, respectively (Figure 1E). Whereas, *trans*-cinnamaldehyde at 20, and 50 μg/mL did not affect biofilm formation of *C. albicans* DAY185 but at 150 μg/mL inhibited biofilm formation by 90% (Figure 1D). However, when *trans*-cinnamaldehyde was tested at 20, 50, and 150 μg/mL, it did not inhibit the biofilm formation of *C. albicans* ATCC 10231 (Figure 1E). Additionally, the biofilm inhibitory effect of *trans*-cinnamaldehyde at 200 μg/mL on *C. albicans* ATCC 10231 was attributed to its antifungal activity (Figures 1C,E). Planktonic cell growths were measured to assess the antifungal activities of α-methyl and *trans*-4-methyl cinnamaldehydes (Figure 1B). Neither of these two analogs inhibited the planktonic growth of *C. albicans* DAY185 or ATCC 10231 at 50 or 100 μg/mL and their MICs were ≥ 200 μg/mL (Figure 1B and Supplementary Figure S1). These results show α-methyl and *trans*-4-methyl cinnamaldehydes effectively prevented biofilm formation by *C. albicans* strains at sub-inhibitory concentrations and that they are more active than *trans*-cinnamaldehyde.

### Cinnamaldehyde Analogs Impaired *C. albicans* Yeast-Hyphae Transition

**FIGURE 2:**
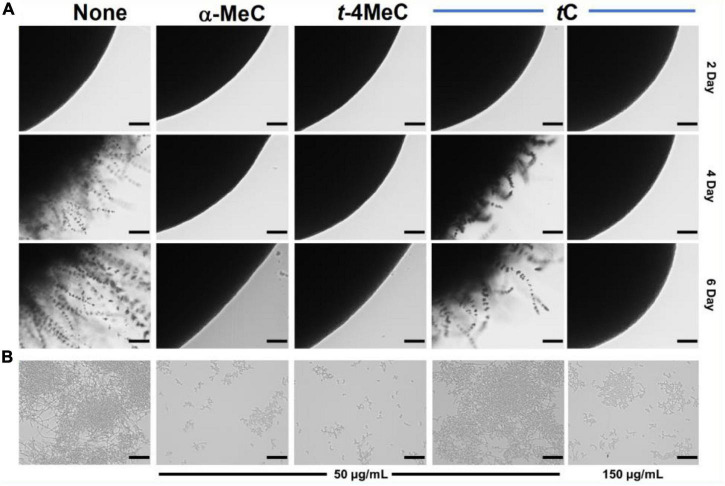
Inhibition of hyphal filamentation and aggregation by α-methyl cinnamaldehyde (α-MeC) and *trans*-4-methyl cinnamaldehyde (*t*-4MeC). *C. albicans* DAY185 was streaked onto PDA solid plates in the absence or presence of α-methyl cinnamaldehyde (α-MeC; 50 μg/mL), *trans*-4-methyl cinnamaldehyde (*t*-4MeC; 50 μg/mL), or *trans*-cinnamaldehyde (*t*C; 50 and 150 μg/mL). Colony morphologies were observed periodically over 6 days at 37°C **(A)**, *C. albicans* DAY185 yeast-hyphae transition was assessed in PDB in the presence of α-methyl cinnamaldehyde (α-MeC; 50 μg/mL), *trans*-4-methyl cinnamaldehyde (*t*-4MeC; 50 μg/mL), or *trans*-cinnamaldehyde (*t*C; 50 and 150 μg/mL) after incubation for 24 h **(B)**. The scale bars in panels **(A,B)** represent 100 μm. None indicates the non-treated control.

A microscopic temporal study of *C. albicans* DAY185 or ATCC 10231 colonies on solid PDA revealed extensive hyphal protrusions. Colonies were monitored over 6 days in the presence or absence of α-methyl or *trans*-4-methyl cinnamaldehydes and compared with *trans*-cinnamaldehyde. In the non-treated control, progressive growth of hyphal filaments was observed. Interestingly, α-methyl and *trans*-4-methyl cinnamaldehydes prevented hyphal protrusions from *C. albicans* DAY185 colonies at 50 μg/mL and revealed colonies with smooth and curved surfaces. However, *trans*-cinnamaldehyde at 50 μg/mL had no effect (Figure 2A), but at 150 μg/mL prevented *C. albicans* DAY185 hyphal protrusions and revealed smooth and curved surfaces. As well as, both the analogs prevented hyphal protrusions from *C. albicans* ATCC 10231 colonies at 20 and 50 μg/mL with colonies surfaces smooth and curved. In contrast, *trans*-cinnamaldehyde at 20, 50, and 150 μg/mL exhibited characteristics similar to the non-treated control of *C. albicans* ATCC 10231 (Supplementary Figures S2A, S3A).

*Candida albicans* biofilm maturation is dependent on a dimorphic switch from yeast to hyphal cells and cell aggregation (Chandra et al., 2001). To examine the effects of α-methyl and *trans*-4-methyl cinnamaldehydes on *C. albicans* morphology, yeast-hyphae transition of *C. albicans* DAY185 or ATCC 10231 was assessed by observing cell aggregation and hyphae formation. After 24 h incubation in PDB medium, large cell aggregates intertwined by hyphae were observed in non-treated controls. At 50 μg/mL both analogs significantly inhibited filamentation and cell aggregation of *C. albicans* DAY185 as compared with that of non-treated controls (Figure 2B). *trans*-cinnamaldehyde had no effect on yeast-hyphae transition at 50 μg/mL, but at 150 μg/mL inhibited the filamentation and aggregation of *C. albicans* DAY185. In case of *C. albicans* ATCC 10231, both analogs at 20 and 50 μg/mL significantly inhibited filamentation and cell aggregation while similar filamentation was observed with *trans*-cinnamaldehyde at 20, 50, and 150 μg/mL. Thus, the *C. albicans* morphology confirms that α-methyl and *trans*-4-methyl cinnamaldehydes inhibit yeast-hypha transition and are more active than *trans*-cinnamaldehyde (Supplementary Figures S2B, S3B).

### Microscopic Examination of *C. albicans* Biofilm Inhibition by α-Methyl and *trans*-4-Methyl Cinnamaldehydes

**FIGURE 3:**
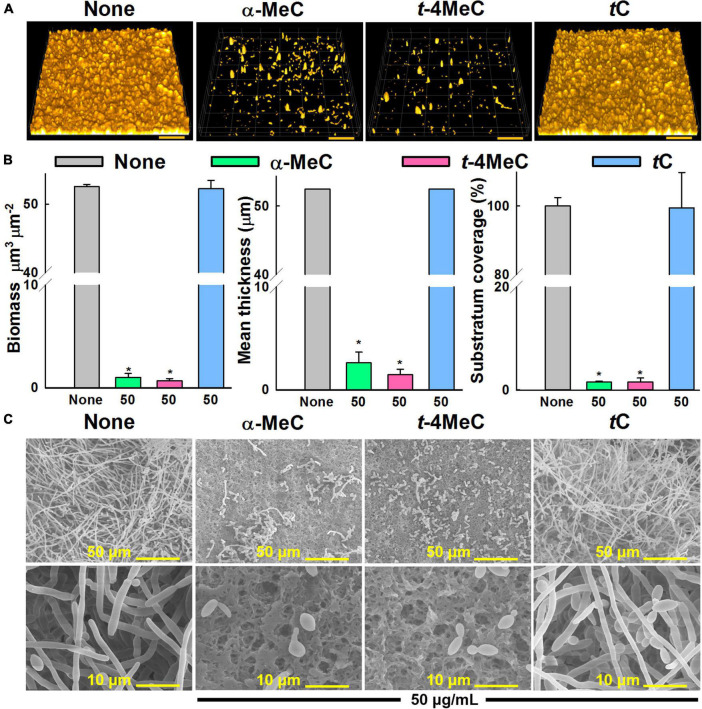
Effect of α-methyl (α-MeC) and *trans*-4-methyl (*t*-4MeC) cinnamaldehydes on *C. albicans* DAY185 biofilms. *C. albicans* DAY185 biofilm formation in the presence of α-methyl cinnamaldehyde (α-MeC), *trans*-4-methyl cinnamaldehyde (*t*-4MeC), or *trans*-cinnamaldehyde (*t*C) at 50 μg/mL was examined by confocal laser microscopy **(A)**, *C. albicans* DAY185 biofilm formations were quantified by COMSTAT analysis **(B)**, *C. albicans* DAY185 biofilm formations on nylon membranes grown in the presence of α-methyl cinnamaldehyde (α-MeC), *trans*-4-methyl cinnamaldehyde (*t*-4MeC), or *trans*-cinnamaldehyde (*t*C) at 50 μg/mL were observed by SEM **(C)**. The scale bars in panel **(A)** represent 100 μm and in panel **(B)** represent 50 and 10 μm. Error bars indicate standard deviations. *P < 0.05 vs. non-treated controls (None). None indicates biofilm formation without treatment after 24 h incubation.

*Candida albicans* biofilm inhibition was analyzed by confocal laser scanning microscopy. In the non-treated control, *C. albicans* formed dense biofilms (thickness > 52 μm and achieved almost 100% surface coverage) after culture for 24 h, whereas the presence of α-methyl or *trans*-4-methyl cinnamaldehydes at 50 μg/mL dramatically reduced biofilm densities and thicknesses. On the other hand, *trans*-cinnamaldehyde at 50 μg/mL had no effect (Figure 3A). Effects on biofilm formation were also measured using COMSTAT biofilm software. Specifically, biofilm biomass, mean thickness, and substrate coverage were reduced by α-methyl and *trans*-4-methyl cinnamaldehydes by > 98% vs. non-treated controls (Figure 3B).

In addition, the antibiofilm activities of α-methyl and *trans*-4-methyl cinnamaldehydes at 50 μg/mL against *C. albicans* DAY185 were examined by SEM. Entirely grown biofilms containing fully formed hyphae were observed on nylon membranes in the absence of cinnamaldehydes (Figure 3C). Interestingly, hyphae formation significantly decreased in the presence of α-methyl or *trans*-4-methyl cinnamaldehydes, whereas no inhibition was observed in the presence of *trans*-cinnamaldehyde (Figure 3C). These observations were in accord with our biofilm formation assay results (Figure 1D). Furthermore, biofilms grown in presence of α-methyl or *trans*-4-methyl cinnamaldehydes had fewer and shorter hyphae and were predominantly composed of yeast and pseudohyphal cells, whereas treatment with *trans*-cinnamaldehyde had no observable effect. Collectively, these results show that α-methyl and *trans*-4-methyl cinnamaldehydes potently inhibit *C. albicans* hyphal formation, cell aggregation, and biofilm formation.

### Gene Expression Changes in *C. albicans* After Treatment by α-Methyl and *trans*-4-Methyl Cinnamaldehydes

**FIGURE 4:**
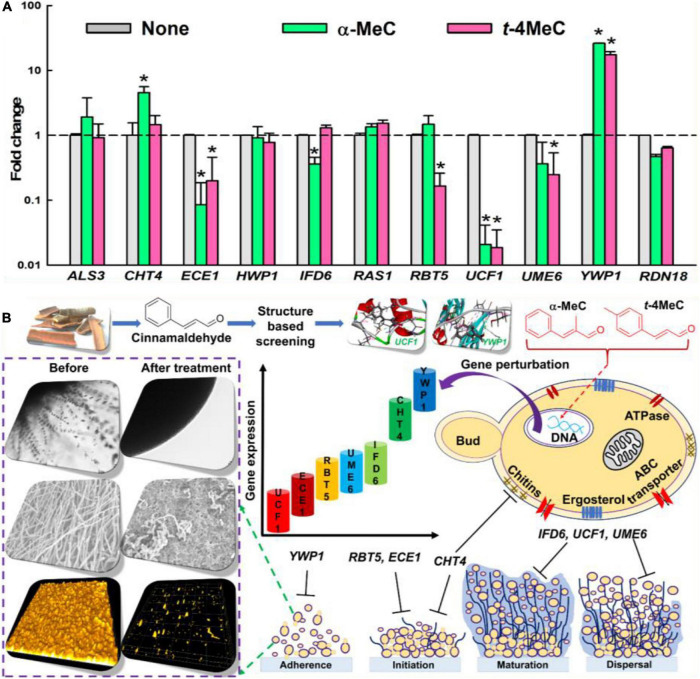
The relative transcriptional profile of ten biofilm- and hypha-related genes. *C. albicans* DAY185 cells incubated with or without α-methyl cinnamaldehyde (α-MeC) or *trans*-4-methyl cinnamaldehyde (*t*-4MeC) at 50 μg/mL for 6 h and relative transcriptional expressions profiles were obtained by qRT-PCR. Fold changes represent alteration in the transcription of treated vs. untreated *C. albicans* DAY185. *RDN18* was used for endogenous normalization of expression levels and the experiment was performed in duplicate (six qRT-PCR reactions were performed per gene) **(A)**. A plausible mechanism of *C. albicans* biofilm inhibition by α-methyl cinnamaldehyde (α-MeC) and *trans*-4-methyl cinnamaldehyde (*t*-4MeC) illustrating all the phenotypic and gene expressional changes **(B).** Error bars indicate standard deviations. *P < 0.05 vs. non-treated controls (None).

qRT-PCR was used to investigate gene expressions of ten biofilm- and hypha-related genes after treating *C. albicans* with α-methyl or *trans*-4-methyl cinnamaldehydes. Transcriptional changes observed after treatment with α-methyl or *trans*-4-methyl cinnamaldehydes at 50 μg/mL were similar (Figure 4A). Notably, the expressions of three key biofilm- and hypha-related genes, namely, *ECE1* (hypha-specific protein, also known as *HWP2*), *UCF1* (filamentous growth), and *UME6* (filament-specific regulator) were repressed by both cinnamaldehyde analogs. For example, α-methyl cinnamaldehyde downregulated *ECE1, IFD6* (alcohol dehydrogenase), and *UCF1* by 12-, 2.8- and 48-fold respectively, and *trans*-4-methyl cinnamaldehyde downregulated *ECE1*, *RBT5* (GPI-modified cell wall protein), *UCF1*, and *UME6* by 5-, 6-, 54-, and 4-fold respectively. While, both α-methyl and *trans*-4-methyl cinnamaldehydes upregulated the expressions of *YWP1* (yeast form wall protein 1) by 26- and 17-fold, respectively (Supplementary Table S2). On the other hand, the expressions of other biofilm and hyphae-related genes (*ALS3*, *HWP1*, and *RAS1*) were unaffected by α-methyl and *trans*-4-methyl cinnamaldehydes. Although α-methyl cinnamaldehyde upregulated *CHT4* (chitinase 4) by 4.6-fold but did not affect *UME6*. In contrast, *trans*-4-methyl cinnamaldehyde had no effect on *CHT4* and *IFD6*. qRT-PCR findings showed that α-methyl and *trans*-4-methyl cinnamaldehydes significantly downregulated biofilm- and hyphae-related genes (i.e., *ECE1, IFD6*, *RBT5*, *UCF1*, and *UME6*) and upregulated biofilm-related genes *CHT4*, and *YWP1* (Figure 4A). Collectively, a plausible mode of action of *C. albicans* biofilm inhibition was depicted to illustrate all of the phenotypic and gene expressional changes caused by α-methyl and *trans*-4-methyl cinnamaldehydes (Figure 4B).

### Molecular Docking of α-Methyl, or *trans*-4-Methyl Cinnamaldehydes With UCF1 or YWP1 to Reveal the Molecular Interaction Profiles

**FIGURE 5:**
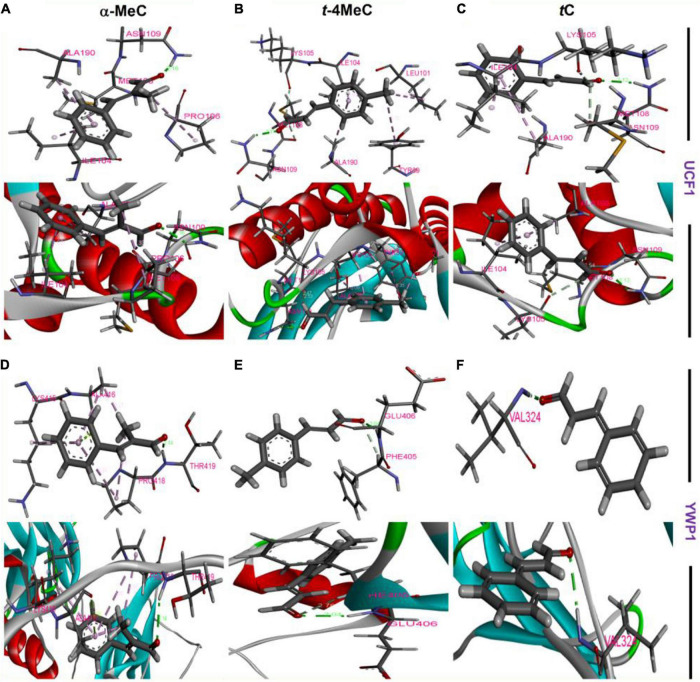
Amino acid residue interactions between UCF1 receptor protein and α-methyl cinnamaldehyde (α-MeC) (A), *trans*-4-methyl cinnamaldehyde (*t*-4MeC) (B), and *trans*-cinnamaldehyde (*t*C) (C), Amino acid residue interactions between YWP1 receptor protein and α-methyl cinnamaldehyde (α-MeC) (D), *trans*-4-methyl cinnamaldehyde (*t*-4MeC) (E), and *trans*-cinnamaldehyde (*t*C) (F).

Molecular dockings were carried out to investigate the molecular interactions between α-methyl and *trans*-4-methyl cinnamaldehydes with amino acid residues of UCF1 and YWP1 proteins, respectively, based on the results obtained in qRT-PCR assay, where, potent antibiofilm agents α-methyl and *trans*-4-methyl cinnamaldehydes, highly downregulated *UCF1* and upregulated *YWP1* genes, respectively (Figure 4A). The binding affinities of α-methyl, *trans*-4-methyl, and *trans*-cinnamaldehydes with the predicted active binding sites of UCF1 and YWP1 fell in the ranges −5.4 to −5.9 kcal/mol and −4.4 to −4.9 kcal/mol, respectively. α-Methyl, *trans*-4-methyl, and *trans*-cinnamaldehydes exhibited binding energies of −5.78, −5.84, and −5.45 kcal/mol, respectively, with the active binding domain of UCF1 (Figures 5A–C and Table 1), and binding energies −4.86, −4.55, and −4.42 kcal/mol, respectively, with the active binding domain of YWP1 (Figures 5D–F and Table 1). α-Methyl, *trans*-4-methyl, and *trans*-cinnamaldehydes formed three π-π, six π-π, or four π-π and one hydrogen bond with Ile104, Pro106, Met108, Asn109, and Ala190; Tyr89, Leu101, Ile104, Lys105, Met108, Asn109, and Ala190; or Ile104, Lys105, Met108, Asn109, and Ala190 amino acid residues of UCF1, respectively (Table 1). Likewise, α-methyl, *trans*-4-methyl, or *trans*-cinnamaldehydes formed four π-π and two hydrogen bonds, one π-π and one hydrogen bond, or one hydrogen bond with Lys415, Ala416, Pro418, Thr419; Phe405, Glu406; or Val324 amino acid residues of YWP1, respectively (Table 1). Also, cluster analysis revealed the number of possible binding positions with UCF1 and YWP1 (Supplementary Figure S4). These qRT-PCR and molecular docking results are compatible for biofilm inhibition since downregulated *UCF1* plays a crucial role in filamentous growth (El Khoury et al., 2018) and upregulated *YWP1* gene has antiadhesive effect and plays a role in biofilm dispersion (Mccall et al., 2019). Overall, the molecular dockings of α-methyl and *trans*-4-methyl cinnamaldehydes were more coherent than that of *trans*-cinnamaldehyde.

**TABLE 1:** Binding energies of targeted ligands with UCF1 or YWP1 proteins.

Compounds	Receptor	Binding energy (Kcal/mol) AUTODOCK	Indicating amino acids	Bonds
α-Methyl cinnamaldehyde	UCF1	−5.7	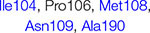	3π–π, 1H
*trans*-4-Methyl cinnamaldehyde	UCF1	−5.84	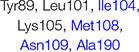	6π–π, 1H
*trans*-Cinnamaldehyde	UCF1	−5.45	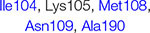	4π–π, 1H
α-Methyl cinnamaldehyde	YWP1	−4.86	Lys415, Ala416, Pro418, Thr419	4π–π, 2H
*trans*-4-Methyl cinnamaldehyde	YWP1	−4.55	Phe405, Glu406	1π–π, 1H
*trans*-Cinnamaldehyde	YWP1	−4.42	Val324	1H

*Amino acid residues essentially required for binding are colored blue.*

### Anthelmintic Activities of the Eleven Cinnamaldehyde Analogs as Determined Using *in vivo* Nematode *C. elegans* Model

**FIGURE 6:**
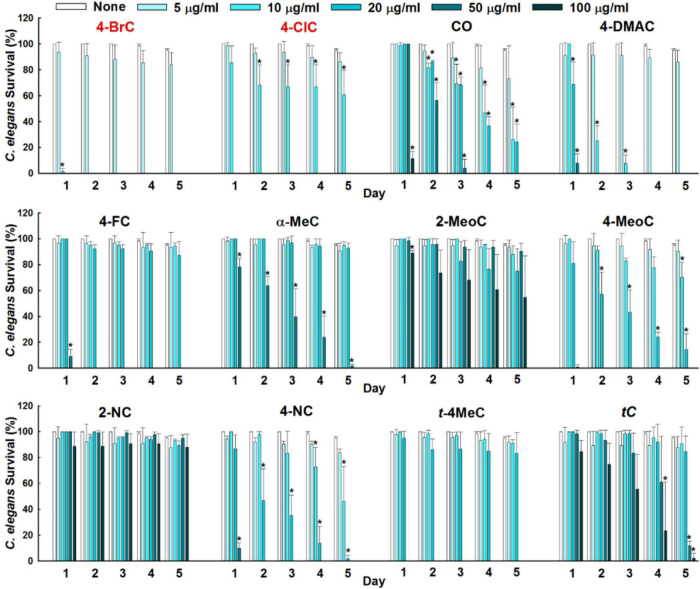
Anthelmintic activities of cinnamaldehyde analogs were evaluated against *C. elegans* for 5 days. Error bars indicate standard deviations. *P < 0.05 vs. non-treated controls (None).

To investigate another possible application for cinnamaldehyde analogs, we investigated their anthelmintic activities using *in vivo C. elegans* model. During initial screening, several cinnamaldehyde analogs, that is, cinnamaldehyde oxime, 4-dimethylamino, 4-fluoro, α-methyl, 4-nitro, and *trans*-4-methyl cinnamaldehydes at 50 μg/mL displayed nematicidal activities by killing all worms over 5 days (Figure 6). While 2-methoxy, 2-nitro, and one positive *trans*-cinnamaldehydes exhibited only minor nematicidal activity at concentrations ≥ 100 μg/mL over 5 days. 4-Bromo and 4-chloro cinnamaldehydes had the most potent nematicidal activities and achieved 100% killing at 10 and 20 μg/mL, respectively, at 2 days of exposure (Figure 6).

### Surface Morphology of *C. elegans* After Treatment With 4-Bromo or 4-Chloro Cinnamaldehydes

Scanning electron microscopy was used to investigate the effects of 4-bromo and 4-chloro cinnamaldehydes on *C. elegans*. 4-Bromo and 4-chloro cinnamaldehydes induced slight structural changes to the cuticle of *C. elegans*. For example, non-treated controls showed smooth circumferential ridges (annuli) and furrows of cuticle, whereas worms treated with 20 μg/mL of 4-bromo or 4-chloro cinnamaldehydes had slightly shriveled cuticles with moderately uniform ridge formations and were shrunken as compared with non-treated controls. *trans*-cinnamaldehyde at 20 μg/mL had little effect on *C. elegans* (Supplementary Figure S5). Taken together both 4-bromo and 4-chloro cinnamaldehydes caused surface morphological changes to the cuticle of *C. elegans*, which confirmed their anthelmintic potentials.

### Cinnamaldehyde Analogs Exhibit Drug-Like Properties

*In silico* pharmacokinetic properties were examined to investigate the possible therapeutic uses of the potent cinnamaldehyde analogs. All potent cinnamaldehyde analogs were scrutinized for compliance with Lipinski’s “rule-of-five” to check their ADME properties. Topological polar surface area (TPSA), molecular weight, lipophilicity, and solubility were examined to predict abilities of drugs to cross membranes. All potent cinnamaldehyde analogs had a log *P* of < 5, a molecular weight < 500, and ≤ 10 hydrogen bond acceptors or ≤ 5 hydrogen bond donors, and a TPSA of < 120 Å^2^. Specifically, 4-bromo, 4-chloro, α-methyl, *trans*-4-methyl or *trans*-cinnamaldehydes had log *P* values in the range 1.9–2.7, molecular weights in the range 130–220 g/moL, one hydrogen bond acceptor and no hydrogen bond donor and a TPSA of 17.07Å^2^ (Supplementary Figure S6 and Supplementary Table S3). Additionally, all potent cinnamaldehyde analogs exhibited an ability to cross the blood-brain barrier and high gastrointestinal absorptions (Supplementary Figure S6 and Supplementary Table S3). Furthermore, none of the potent cinnamaldehyde analogs violated Lipinski’s “rule-of-five,” which suggested all have therapeutic potential.

## Discussion

Based on structure similarities, we screened out cost-effective cinnamaldehyde analogs that inhibit biofilm formation rather than cell growth to reduce the risk of drug-resistance development. Remarkably, the substitution of methyl on aromatic ring of cinnamaldehyde may be responsible for the potent antibiofilm effect. As previously reported (Brackman et al., 2011) that electron withdrawing group enhanced the activity of cinnamaldehyde analogs, we speculate that our cinnamaldehyde analogs may work in the similar manner. Furthermore, presence of α, β-unsaturated carbonyl pharmacophore in cinnamaldehyde analog structures may serve as biological essential group (Chen et al., 2017). These electrophilic acceptors could react with nucleophiles by Michael type addition resulting in cinnamaldehyde analogs-receptor conjugates which probably suggest potent activities of the cinnamaldehyde. Meanwhile, α-methyl and *trans*-4-methyl cinnamaldehydes at sub-MIC concentrations inhibited biofilms formation more than *trans*-cinnamaldehyde by inhibiting hyphae formation. Hyphae assays and SEM results showed that α-methyl and *trans*-4-methyl cinnamaldehydes inhibited *C. albicans* filamentation markedly more than *trans*-cinnamaldehyde and that observed in non-treated controls (Figures 2, 3C). Interestingly, *trans*-cinnamaldehyde at 50 μg/mL had no effect on biofilm formation by *C. albicans* DAY185 or ATCC 10231, which contrasts with reports (Ying et al., 2019; Miranda-Cadena et al., 2021) that it is effective against different strains of *C. albicans*, and suggests *trans*-cinnamaldehyde inhibits biofilm formation at higher concentrations. These results concur with the findings of Taguchi et al. (2013), who concluded cinnamaldehyde acts against *C. albicans* in two different ways, that is, by inhibiting mycelial growth and killing activity by causing membrane damage. *trans*-cinnamaldehyde has also been reported to disrupt the activities of mitochondria, cell-wall synthesizing enzyme β-1-3-glucan, and chitin in other organisms (Bang et al., 2000; Ka et al., 2003).

Interestingly, our qRT-PCR studies showed that the down-regulations of *ECE1*, *IFD6*, *RBT5*, *UCF1*, and *UME6* in *C. albicans* cells by α-methyl and *trans*-4-methyl cinnamaldehydes (Figure 4A). Specifically, *ECE1* is essential for hyphal development and their expressions have been shown to be correlated with cell elongation and biofilm formation (Lee et al., 2021). *RBT5* which putatively encodes GPI-modified cell wall protein (Perez et al., 2006). *IFD6* negatively affects the matrix production (Nobile et al., 2009). *UCF1* plays major role in filamentous growth and *UME6* is a filament specific regulator of *C. albicans* hyphal extension and enhances the biofilm formation (Banerjee et al., 2013; El Khoury et al., 2018). Moreover, *CHT4*, and *YWP1* genes were upregulated in favor of biofilm inhibition, as upregulation of *CHT4* suggested the degradation of chitin in cell wall (Drakulovski et al., 2011) and *YWP1* has antiadhesive effect and plays a role in biofilm dispersion (Mccall et al., 2019). It has been reported that *C. verum* essential oils significantly downregulated the expressions of another set of biofilm or hypha related genes, namely, *RAS*, *EFG1*, *CYR*, *CPH*, *HWP1*, *ALS3*, *SAP2*, *SAP4*, *SAP5*, *SAP6*, and *HST7*, and up-regulated *NRG* in *C. albicans* (Essid et al., 2019). Also, Khan et al. (2017) reported cinnamaldehyde downregulated *HWP1* expression, whereas we found cinnamaldehyde treatment did not affect the expressions of *RAS*, *ALS3*, or *HWP1*. Consequently, it is confirmed that treatments with α-methyl and *trans*-4-methyl cinnamaldehydes inhibited biofilm formation of *C. albicans* by inducing phenotypic and gene expressional changes *via* hyphal growth inhibition.

The conformation interaction of UCF1 with α-methyl, and *trans*-4-methyl cinnamaldehydes showed that common amino acid residues such as Ile104, Met108, Asn109, and Ala190 are essential for the molecular interaction. Also, α-methyl, and *trans*-4-methyl cinnamaldehydes with YWP1 protein formed the conformation interaction with Lys415, Ala416, Pro418, Thr419, Phe405, Glu406, and Val324 amino acid residues. Gene expression and molecular interaction studies revealed that α-methyl, and *trans*-4-methyl cinnamaldehydes might work as antibiofilm agents *via* inhibiting filament and suppressing adhesive effect of *C. albicans*.

Cinnamaldehyde and its analog cinnamaldehyde oxime are well-known nematicides and natural alternatives to synthetic anthelmintic agents and pesticides against the animal and plant parasites *Ascaris suum* and *Meloidogyne incognita* (Williams et al., 2015; Ferreira Barros et al., 2021). According to our findings, cinnamaldehyde analogs have anthelmintic activity and 4-bromo and 4-chloro cinnamaldehydes were the most active at 10 and 20 μg/mL, respectively. Lu et al. (2020) suggested that cinnamaldehyde disrupts glutathione metabolism and found that at 800 μg/mL caused 100% *C. elegans* mortality, which is several fold higher than the mortality rates observed for the cinnamaldehyde analogs tested in the present study (5–100 μg/mL). Slight surface morphological changes to the cuticle of *C. elegans* by 4-bromo and 4-chloro cinnamaldehydes confirmed their anthelmintic effects. Ropiak et al. (2016) reported that higher concentrations (2 mM) of *trans*-cinnamaldehyde are required to cause morphological changes to *C. elegans* cuticles. Also, Williams et al. (2015) reported marked damage to digestive tissues and the muscular layer in *A. suum* after treatment with cinnamaldehydes as a anthelmintic agents. Besides, Brackman et al. (2011) reported that *trans*-cinnamaldehyde has IC_50_ value of 77 μM against MRC-5 cells, which suggests that the therapeutic window of cinnamaldehyde analogs may be high enough for therapeutic applications in humans and animals. Moreover, Lipinski’s “rule-of-five” summarizes vital molecular pharmacokinetic properties of compounds that predict their potential applicability’s as oral drugs (Benet et al., 2016). All potent cinnamaldehyde analogs examined exhibited drug-like properties and did not violate the “rule-of-five” (Supplementary Figure S6). These tested cinnamaldehyde analogs may work well toward human cell lines; therefore, it is needed to test their toxicity toward *in vivo* models.

Thus, our observations suggest α-methyl and *trans*-4-methyl cinnamaldehydes can be used as a potential molecule for further drug discovery as multitargets antibiofilm molecules and that 4-bromo and 4-chloro cinnamaldehydes might be used as anthelmintic agents for the development of new therapeutic candidates.

## Conclusion

Novel antivirulence agents are required to address the challenges posed by drug-resistant microorganisms. Our *in vitro* studies collectively showed that α-methyl and *trans*-4-methyl cinnamaldehydes inhibited *C. albicans* biofilm formation without killing *C. albicans*. Also, our potent cinnamaldehyde analogs act as suppressors of the *UCF1* and *YWP1* genes in *C. albicans*. These compounds can be considered to treat persistent infections either singly or in combination or as adjunctive treatments. In addition, 4-bromo and 4-chloro cinnamaldehydes exhibited potent anthelmintic effects and can be used as anthelmintic agents. Hence, the present study demonstrates cinnamaldehyde analogs can serve as potential molecules to provide a basis to design effective drug molecules for the treatment of multidrug-resistant microbial agents causing human infections.

## Data Availability Statement

The original contributions presented in the study are included in the article/Supplementary Material, further inquiries can be directed to the corresponding author/s.

## Author Contributions

SK and JL: conceptualization. SK, J-HL, and VR: methodology. SK and VR: software. SK, VR, J-HL, and Y-GK: validation and formal analysis and investigation. JL: resources. SK, VR, and JL: data curation, writing of the manuscript, and visualization. J-HL and JL: project administration. All authors have read and agreed to the published version of the manuscript.

## Conflict of Interest

The authors declare that the research was conducted in the absence of any commercial or financial relationships that could be construed as a potential conflict of interest.

## Publisher’s Note

All claims expressed in this article are solely those of the authors and do not necessarily represent those of their affiliated organizations, or those of the publisher, the editors and the reviewers. Any product that may be evaluated in this article, or claim that may be made by its manufacturer, is not guaranteed or endorsed by the publisher.

## Footnotes

1 https://www.uniprot.org/uniprot/Q59KG2 and https://www.uniprot.org/uniprot/Q59Y31, respectively, accessed 1 September 2021.
