# Supplementary material for: Appraisal of Cinnamaldehyde Analogs as Dual-Acting Antibiofilm and Anthelmintic Agents
Source: Front Microbiol. 2022 Mar 16;13:818165. doi: 10.3389/fmicb.2022.818165 (PMC8966877; doi:10.3389/fmicb.2022.818165)
Supplement: Supplementary file 1 [file Data_Sheet_1.docx]

**Supplementary Data**

**Appraisal of cinnamaldehyde analogues as dual-acting antibiofilm and anthelmintic agents**

Sagar Kiran Khadke^†^, Jin-Hyung Lee^†^, Yong-Guy Kim, Vinit Raj, Jintae Lee*

*School of Chemical Engineering, Yeungnam University, Gyeongsan, 38541, Republic of Korea*

^*^**Corresponding author:** E-mail: [jtlee@ynu.ac.kr](mailto:jtlee@ynu.ac.kr)

Phone: 82-53-810-2533, Fax: 82-53-810-4631

^†^ - These authors have contributed equally to this work.

**Supplementary Table S1. Primer sequences used for qRT-PCR.**

| ***Gene*** | ***Primer*** |
| --- | --- |
| ***ALS3*** | *Forward 5'-CAA CAT CAA CCA ACC AAT CTC-3'* |
|  | *Reverse 5'-TGA ATA ACA GAA CCA GAT CCG-3'* |
| ***CHT4*** | *Forward 5'-GTA CGA TTG AAT TTG CTG AG-3'* |
|  | *Reverse 5'-TTG GAT GAA CTC CCT TGT TA-3'* |
| ***ECE1*** | *Forward 5'-CCA GAA ATT GTT GCT CGT GTT GCC A-3'* |
|  | *Reverse 5'-TCC AGG ACG CCA TCA AAA ACG TTA G-3'* |
| ***HWP1*** | *Forward 5'-TTG TTT GCG TCA TCA AGA CTT TG-3'* |
|  | *Reverse 5'-GTC TTC ATC AGC AGT AAC ACA ACC A-3'* |
| ***IFD6*** | *Forward 5'-TTG GGA AGA TTT TGA TCC TGT TG-3'* |
|  | *Reverse 5'-CGA GTG CAT GAT TTC TTC ATA AGT G-3'* |
| ***RAS1*** | *Forward 5'-* *GGC CAT GAG AGA ACA ATA TA-3'* |
|  | *Reverse 5'-* *GTC TTT CCA TTT CTA AAT CAC-3'* |
| ***RBT5*** | *Forward 5'-CTG CTG AAA GTT CTG CAC CA-3'* |
|  | *Reverse 5'-GCT TCA ACG GAA ACA GAA GC-3'* |
| ***UCF1*** | *Forward 5'-ATG GCG GGA AAG AAA AAG TC-3'* |
|  | *Reverse 5'-CCC AAG TTT CAT CAC GAA CA-3'* |
| ***UME6*** | *Forward 5'-AGC ACC AAA TTC GCC TTA TG-3'* |
|  | *Reverse 5'-AGG TTG AGC TTG CTG CAG TT-3'* |
| ***YWP1*** | *Forward 5'-GTT CCA TTT TTC CAA GTT CAT TTA G-3'* |
|  | *Reverse 5'-TCA AGA GTA GAA CCT TCA AGA GCA G-3'* |
| ***RDN18*** | *Forward 5'-AGA AAC GGC TAC CAC ATC CCA-3'* |
|  | *Reverse 5'-CGA ATG GGC CCT GTA TCG T-3'* |

**Supplementary Table S2. Genes down/up – regulation by cinnamaldehyde analogues.**

| **Gene** | ***α*-methyl cinnamaldehyde** | ***trans*-4-methyl cinnamaldehyde** | ***trans* -cinnamaldehyde** |
| --- | --- | --- | --- |
| **Downregulation** | *ECE1, IFD6, UCF1,* | *ECE1, RBT5, UCF1, UME6* | *RAS1, EFG1, CYR1, CPH1, HST, HWP, ALS3, SAP2, SAP4, SAP5, SAP6,* |
| **Upregulation** | *CHT4, YWP1* | *YWP1* | *NRG* |
| **References** | This study | This study | ([Khan et al., 2017](#_ENREF_2); [Essid et al., 2019](#_ENREF_1)) |

**Supplementary Table S3.** Various physicochemical parameters of potent cinnamaldehyde analogues to reveal the possible ADME properties.

| **Parameters** | **4-Bromo cinnamaldehyde** | **4-Chloro cinnamaldehyde** | ***α*-Methyl cinnamaldehyde** | ***trans*-4-Methyl cinnamaldehyde** | ***trans*-Cinnamaldehyde** |
| --- | --- | --- | --- | --- | --- |
| **Formula** | C_9_H_7_BrO | C_9_H_7_ClO | C_10_H_10_O | C_10_H_10_O | C_9_H_8_O |
| **Molecular Weight (g/moL)** | 211.06 | 166.60 | 146.19 | 146.19 | 132.16 |
| **H-bond acceptors** | 1 | 1 | 1 | 1 | 1 |
| **H-bond donors** | 0 | 0 | 0 | 0 | 0 |
| **Topological Polar Surface Area (Å^2^)** | 17.07 | 17.07 | 17.07 | 17.07 | 17.07 |
| **LogP** | 2.65 | 2.42 | 2.25 | 2.31 | 1.97 |
| **Gastrointestinal absorption** | High | High | High | High | High |
| **Blood Brain Barrier permeability** | Yes | Yes | Yes | Yes | Yes |
| **Lipinski violations** | 0 | 0 | 0 | 0 | 0 |
| **Bioavailability Score** | 0.55 | 0.55 | 0.55 | 0.55 | 0.55 |
| **CYP2C19 inhibitor** | No | No | No | No | No |

**
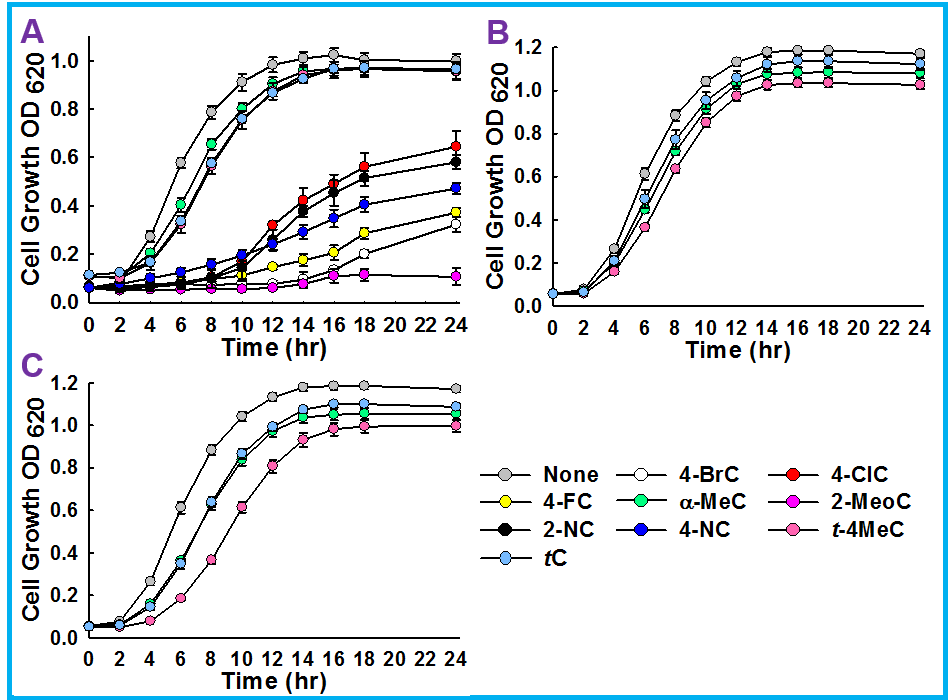
**

**Supplementary Figure S1**. *C. albicans* DAY185 cell growth was investigated in the presence of eight antibiofilm cinnamaldehyde analogues and *trans*-cinnamaldehydes at 50 μg/mL (**A**). *C. albicans* ATCC 10231 cell growth was investigated in the presence of *α*-methyl cinnamaldehyde (*α*-MeC), *trans*-4-methyl cinnamaldehyde (*t*-4MeC) and *trans*-cinnamaldehyde (*t*C) at 50 μg/mL (**B**), and at 100 μg/mL (**C**). Error bars indicate standard deviations.


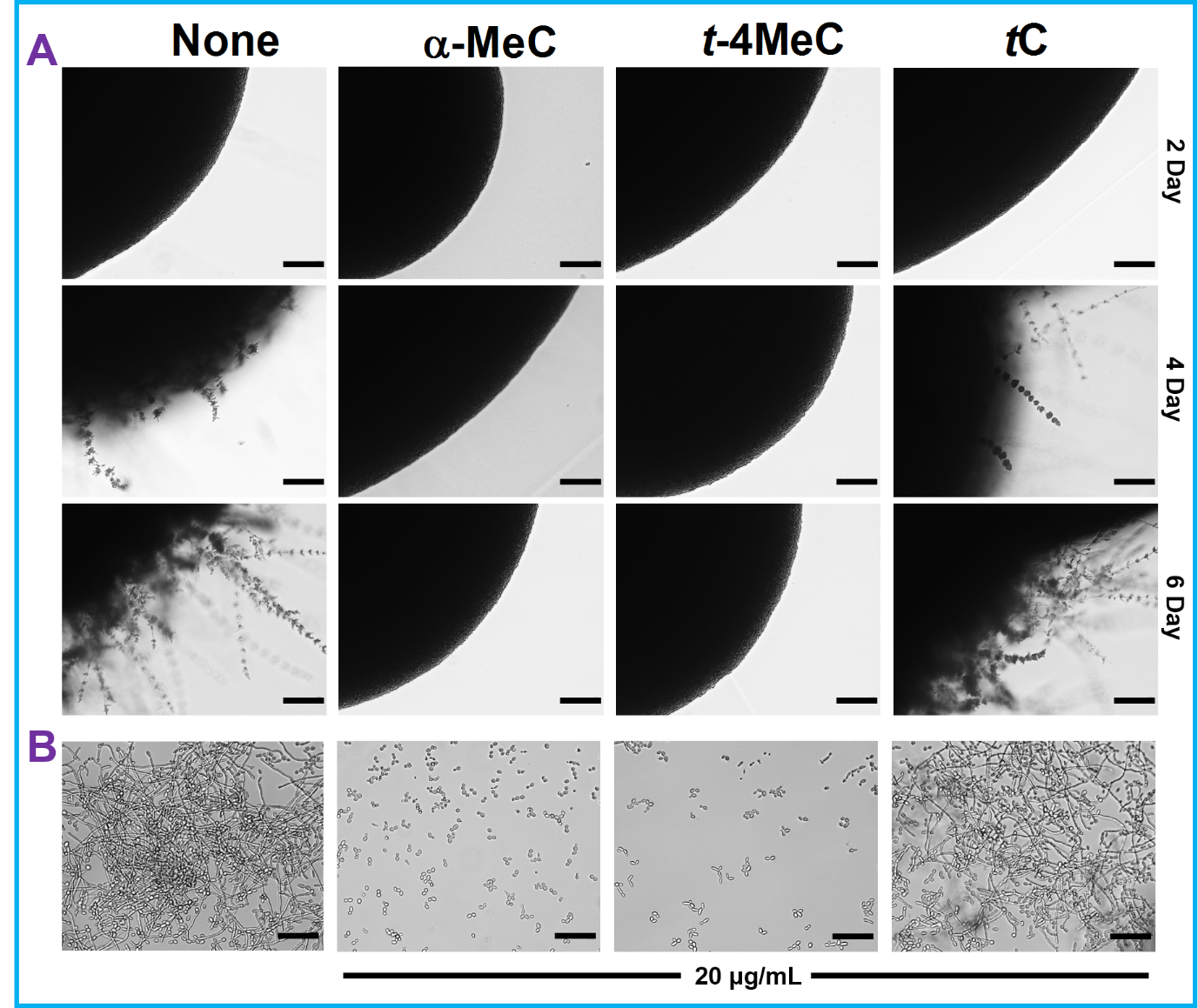


**Supplementary Figure S2**. Inhibition of hyphal filamentation and aggregation by *α*-methyl cinnamaldehyde (*α*-MeC) and *trans*-4-methyl cinnamaldehyde (*t*-4MeC). *C. albicans* ATCC 10231 was streaked onto PDA solid plates in the absence or presence of *α*-methyl cinnamaldehyde (*α*-MeC), *trans*-4-methyl cinnamaldehyde (*t*-4MeC), or *trans*-cinnamaldehyde (*t*C) at 20 μg/mL. Colony morphologies were observed periodically over 6 days at 37 °C **(A)**, *C. albicans* ATCC 10231 yeast-hyphae transition was assessed in PDB in the presence of *α*-methyl cinnamaldehyde (*α*-MeC), *trans*-4-methyl cinnamaldehyde (*t*-4MeC), or *trans*-cinnamaldehyde (*t*C) at 20 μg/mL after incubation for 24 h **(B)**. The scale bars in panels A and B represent 100 µm. None indicates the non-treated control.


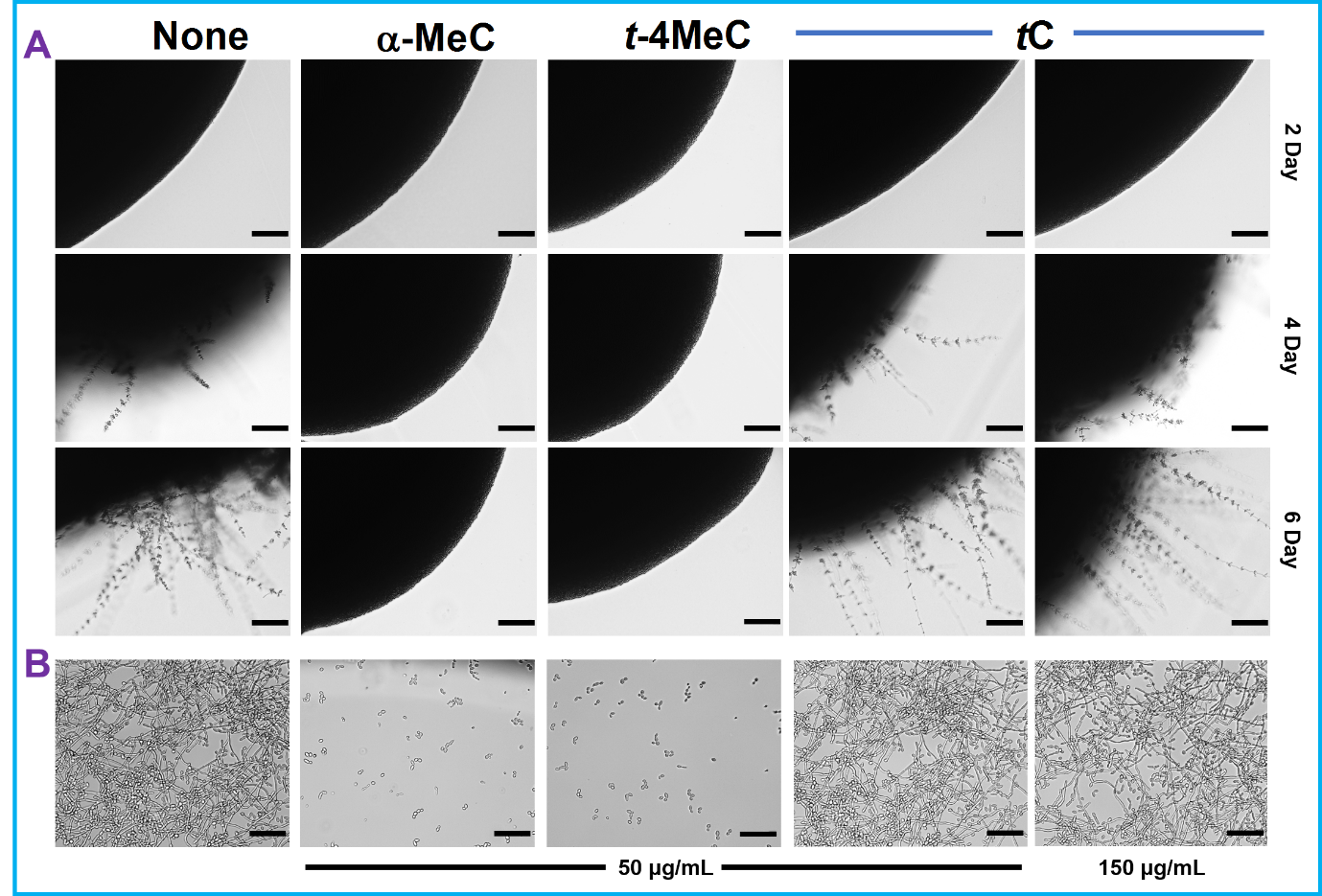


**Supplementary Figure S3**. Inhibition of hyphal filamentation and aggregation by *α*-methyl cinnamaldehyde (*α*-MeC) and *trans*-4-methyl cinnamaldehyde (*t*-4MeC). *C. albicans* ATCC 10231 was streaked onto PDA solid plates in the absence or presence of *α*-methyl cinnamaldehyde (*α*-MeC; 50 μg/mL), *trans*-4-methyl cinnamaldehyde (*t*-4MeC; 50 μg/mL), or *trans*-cinnamaldehyde (*t*C; 50 and 150 μg/mL). Colony morphologies were observed periodically over 6 days at 37 °C **(A)**, *C. albicans* ATCC 10231 yeast-hyphae transition was assessed in PDB in the presence of *α*-methyl cinnamaldehyde (*α*-MeC; 50 μg/mL), *trans*-4-methyl cinnamaldehyde (*t*-4MeC; 50 μg/mL), or *trans*-cinnamaldehyde (*t*C; 50 and 150 μg/mL) after incubation for 24 h **(B)**. The scale bars in panels A and B represent 100 µm. None indicates the non-treated control.


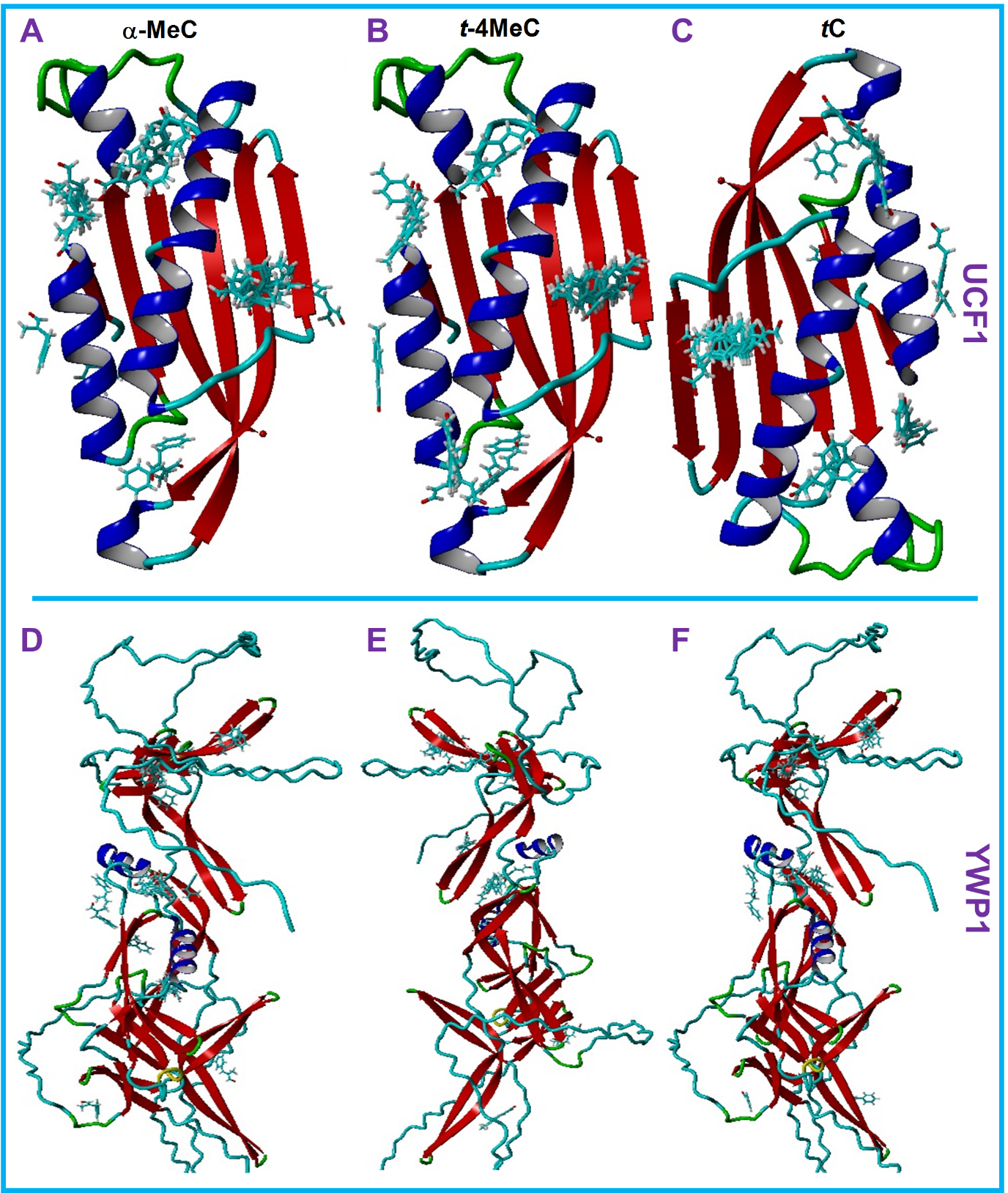


**Supplementary Figure S4**. Cluster analysis of UCF1 receptor protein with *α*-methyl cinnamaldehyde (*α*-MeC) (**A**), *trans*-4-methyl cinnamaldehyde (*t*-4MeC) (**B**), and *trans*-cinnamaldehydes (*t*C) (**C**), Cluster analysis of YWP1 receptor protein with *α*-methyl cinnamaldehyde (*α*-MeC), (**D**), *trans*-4-methyl cinnamaldehyde (*t*-4MeC) (**E**), and *trans*-cinnamaldehyde (*t*C) (**F**).

**
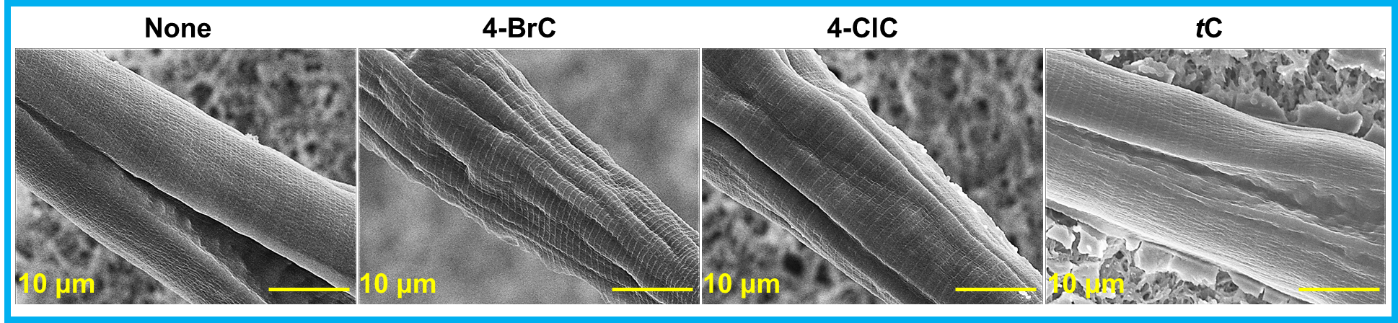
**

**Supplementary Figure S5**. *C. elegans* cuticle was observed by SEM in the presence of 4-bromo cinnamaldehyde (4-BrC), 4-chloro cinnamaldehyde (4-ClC), or *trans*-cinnamaldehyde (*t*C) at 20 μg/mL. The scale bar represents 10 µm.


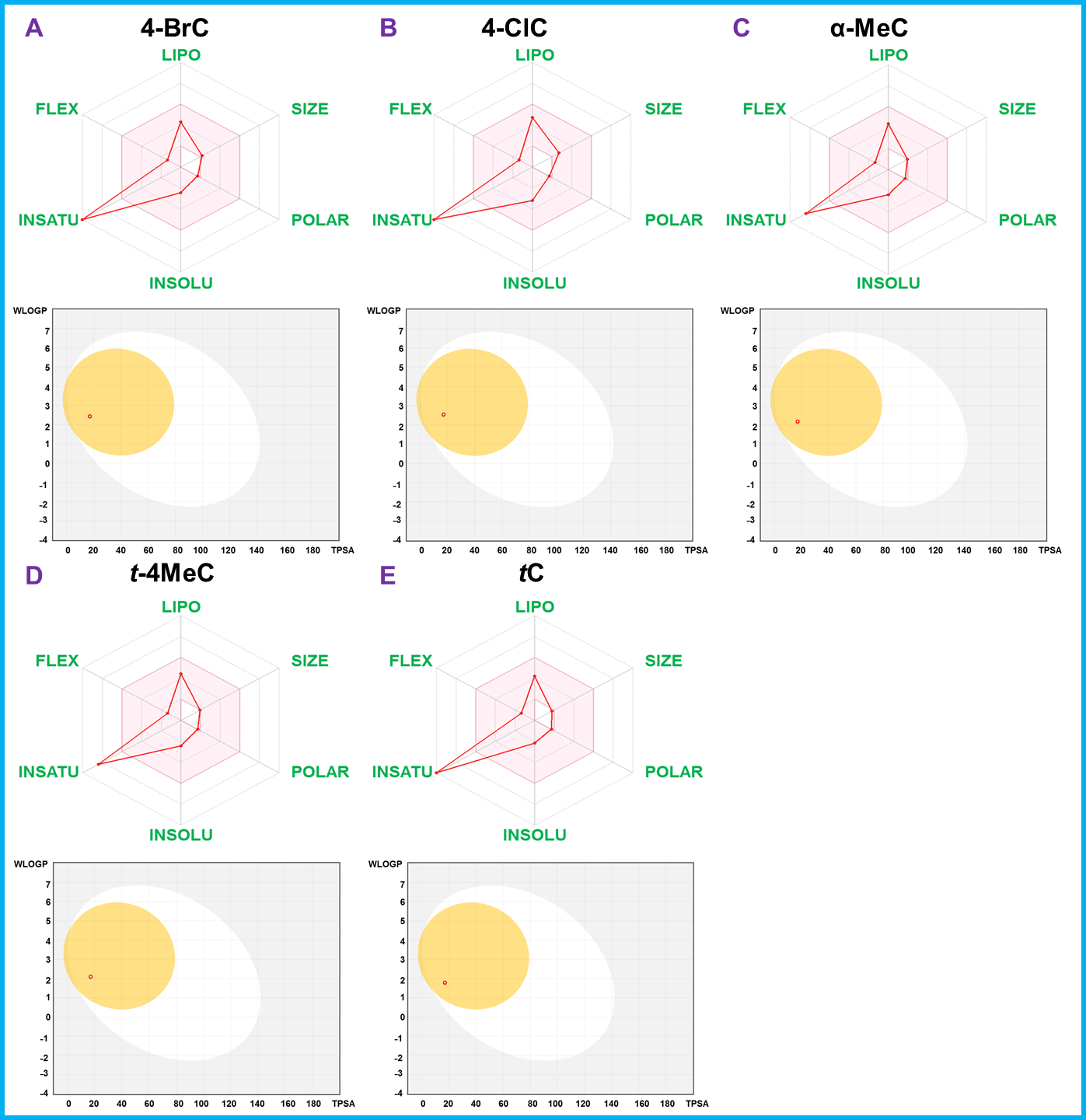


**Supplementary Figure S6**. ADME and BOILED-Egg analysis for the selected cinnamaldehyde analogues was investigated using Swiss ADME server to calculate drug physicochemical properties such as lipophilicity (LIPO), size, polarity (POLAR), insolubility in water (INSOLU), instauration (INSATU), and flexibility (FLEX) and intuitive evaluation of passive gastrointestinal absorption (HIA) and brain penetration (BBB) function in the WLOGP-*versus*-TPSA referential respectively of 4-bromo cinnamaldehyde (4-BrC) (**A**), 4-chloro cinnamaldehyde (4-ClC) (**B**), *α*-methyl cinnamaldehyde (*α*-MeC) (**C**) *trans*-4-methyl cinnamaldehyde (*t*-4MeC) (**D**), and *trans*-cinnamaldehyde (*t*C) (**E**).

**References**

Essid, R., Gharbi, D., Abid, G., Karkouch, I., Hamouda, T.B., Fares, N., Trabelsi, D., Mhadhbi, H., Elkahoui, S., Limam, F., and Tabbene, O. (2019). Combined effect of *Thymus capitatus* and *Cinnamomum verum* essential oils with conventional drugs against *Candida albicans* biofilm formation and elucidation of the molecular mechanism of action. *Industrial Crops and Products* 140**,** 111720.

Khan, S.N., Khan, S., Iqbal, J., Khan, R., and Khan, A.U. (2017). Enhanced killing and antibiofilm activity of encapsulated cinnamaldehyde against *Candida albicans*. *Front Microbiol* 8**,** 1641.
